# Production of Recombinant Adenovirus Containing Human Interlukin-4 Gene

**Published:** 2011

**Authors:** Majid Mojarrad, Yassan Abdolazimi, Jamshid Hajati, Mohammad Hossein Modarressi

**Affiliations:** 1 *Department of Medical Genetics, Faculty of Medicine, Mashhad University of Medical Sciences, Mashhad, Iran*; 2 *Department of Medical Genetics, Faculty of Medicine, Tehran University of Medical Sciences, Tehran, Iran*; 3 *Department of Immunology, Faculty of Medicine, Tehran University of Medical Sciences, Tehran, Iran*

**Keywords:** Cloning, Interlukin-4, Recombinant Adenovirus

## Abstract

**Objective(s):**

Recombinant adenoviruses are currently used for a variety of purposes, including *in vitro* gene transfer, *in vivo* vaccination, and gene therapy. Ability to infect many cell types, high efficiency in gene transfer, entering both dividing and non dividing cells, and growing to high titers make this virus a good choice for using in various experiments. In the present experiment, a recombinant adenovirus containing human IL-4 coding sequence was made. IL-4 has several characteristics that made it a good choice for using in cancer gene therapy, controlling inflammatory diseases, and studies on autoimmune diseases.

**Materials and Methods:**

In brief, IL-4 coding sequence was amplified by and cloned in pAd-Track-CMV. Then, by means of homologous recombination between recombinant pAd-Track-CMV and Adeasy-1 plasmid in bacteria, recombinant adenovirus complete genome was made and IL-4 containing shuttle vector was incorporated into the viral backbone. After linearization, for virus packaging, viral genome was transfected into HEK-293 cell line. Viral production was conveniently followed with the aid of green fluorescent protein.

**Results:**

Recombinant adenovirus produced here, was capable to infecting cell lines and express interlukin-4 in cell.

**Conclusion:**

This system can be used as a powerful, easy, and cost benefit tool in various studies on cancer gene therapy and also studies on immunogenetics.

## Introduction

Recombinant Adenovirus vectors have been extensively used to deliver foreign genes to a variety of cell types and tissues both *in vitro* and *in vivo* (1,2). They can be easily grown to high titer and can efficiently transfer genes into both dividing and non dividing cells. These properties make them not only promising vectors for gene therapy and functional studies, but also important tools for gene transfer into mammalian cells. 

One of the genes that recently became interesting in gene therapy researches is human interleukin 4 (hIL-4). hIL-4, which was co-discovered by Maureen, William and Vitetta in 1982 (3), is a cytokine produced by activated CD4+ T lymphocytes. It has pluripotent activities such as stimulation of T-cell and activated B-cell proliferation, differentiation of CD4+ T-cells into Th2 cells (3), induction of IFN-gamma production, and expression of IgE and IgG4 in human B cells (4).

A single IL-4 gene per haploid genome exists on human chromosome 5 in region q31(5), and codes for a protein with 129 amino acids, including two possible sites for N-linked glycosylation and 6-cystein residues, which form three disulfide bonds (6).

 IL-4 has a wide rang of application in immunotherapy against bacterial infections (e.g. *Borrelia burgdorferi* infection) (7), parasitic infections (e.g. *Schistosoma japonicum* infection) (8) and even cancer (e.g. malignant astrocytoma, glioma, glioblastoma and ) (9-12). Since 1997 that IL-4 adenovirus was used in a study on type I immunity responses, several different groups have employed this system for different purposes (13-15). 

In this report, we attempted to optimize recombinant adenovirus production process and also produce a recombinant adenovirus expressing hIL-4, in order to effectively express IL-4 in cells. 

## Materials and Methods

***Samples and cell lines***

Bone marrow samples were provided as described previously (16). Total RNA was extracted from bone marrow samples by TriPure RNA extraction kit and single-stranded cDNA was prepared from 5 μg total RNA using MMLV reverse transcriptase and random hexamer (Fermentas) according to manufacturer’s instruction. Briefly, 1 µg of RNA was RT into cDNA in a 20 µl reaction for 10 min at 25 °C and 60 min at 42 °C using 25 ng/µl random hexamer, 20 U of RNase inhibitor, 25 µM dNTP and 200 U of MMLV-RT according to the manufacturer’s instructions.

Human embryonic kidney cells (HEK293) were cultured in RPMI 1640 (Boisera) containing 20 µg/ ml gentamicine supplemented with 10% FBS (Invitrogen, Carlsbad, CA). Cells were grown at 37 °C, in presence of 5% CO_2_ and 95% humidity.

***Plasmid, bacterial strain and reagents***

pAd-Track-CMV and Ad-Easy1 plasmids were a gift from TONG-CHUAN HE (17). Xl Blue and BJ5183 *Escherichia coli* strains were used as bacterial hosts. T4 ligation kit, RNA extraction kit, cDNA synthesis kit, restriction endonuclease, and chemical reagents were purchased from Roche (Germany).

" extraction from gel" kit was purchased from Fermentas (Lithuania). All primers were designed according to sequences available on genomic databases. Extraction of plasmid was done by alkaline lysis procedure. Molecular techniques were performed according to manufacturer’s instruction. Transformation was performed based on standard methods with some modifications (18).

***Amplification of IL4 open reading frame (ORF)***

cDNA samples were checked for their quality using primers designed from exon 10 5′-TCCgACTgAGCggCACTgggAgTgC-3′ and exon 11 5′-gCCCGCAggTCCTCTTTCCCTCACA-3′ of the housekeeping gene phosphoglucomutase-1 (*PGM1*) (gene bank accession NO. NM_002633). The RT- amplification was carried out in a 25-μl reaction mixture containing 1x buffer (50 mM KCL, 50 mM Tris-HCl, pH= 8.4, and 1.5 mM of MgCl_2_), each dNTP at a concentration of 50 µM, each primer at a concentration of 0.4 µM, and 1 unit of Taq DNA polymerase enzyme. One μl cDNA was added to reaction mixture. Reaction tubes were placed in Eppendorf thermal cycler (Germany) programmed for 30 cycles of denaturation at 94 °C for 45 sec, 45 sec of annealing at 64 °C, and 50 sec of extension at 72 °C; an additional 3 min of denaturation at 94 °C preceded the first cycle and elongation was extended to 7 min in the end. Ten µl of products were run on 2% agarose gel (Invitrogen, UK), stained with ethidium bromide (EtBr), and then photographed under ultraviolet light.

Positive samples were used for IL-4 amplification. A pair of cloning primers (forward—HILf: 5′- CCgCTCgAgATgggTCTCACCTCCCAACTg-3′, reverse HILr: 5′ CCgCTCgAgTATTCAgCTCgAACACTTTg -3′), were designed to amplify a 467-bp open reading frame of IL-4 (gene bank accession NO. NM_000589). PCR reaction was performed using Pfu DNA polymerase enzyme (Fermentas) and amplification was performed in the same reaction mixture as described above except for IL-4 specific primers. reactions consisted of preliminary denaturation at 95 °C for 3 min followed by 35 cycles of 95 °C for 30 sec, 55.7 °C for 30 sec and 72 °C for 45 sec followed by a 7-min final extension at 72 °C. Ten µl of products were run on 2% agarose gel (Invitrogen, UK), stained with EtBr, and then photographed under ultraviolet light.

PCR products were digested using *EcoRV* and *PstI*. Digestion mixtures contained 5 μl products, 4 units of enzyme, and 1 μl10× digestion buffer, and H_2_O to a final volume of 10 μl. Reactions were allowed to proceed for 2 hr at 37 °C and digestion results were estimated by agarose gel electrophoresis.

***Cloning of IL-4 PCR product into pAd-Track-CMV vector***

 products were digested by *XhoI* enzyme (Roche). Digestion mixtures contained 30 μl products, 12 units of *XhoI* enzyme, 4 μl of the enzyme 10X buffer, and H_2_O to total volume of 40 μl. The reactions were allowed to proceed for 20 hr at 37 °C.

**Figure 1.**
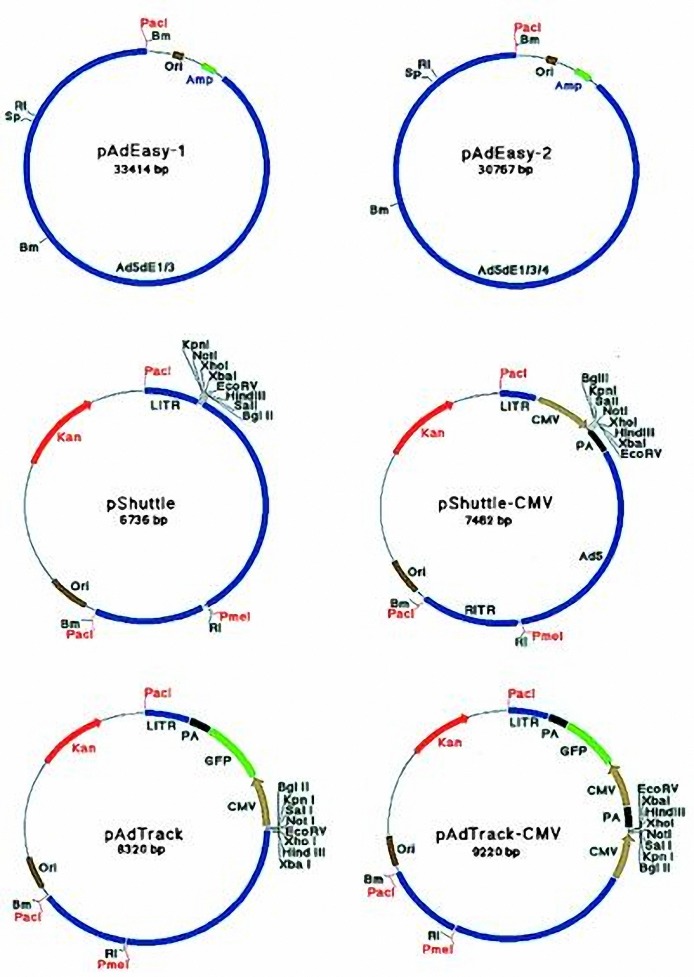
pAd-Track-CMV plasmid map (17)

Digestion products were run on 2% agarose gel and digested band was cut and extracted by extraction from gel kit following the manufacturer's instruction. Finally, concentration of recovered was estimated by biophotometer. *XhoI *containing plasmid (17), pAd-Track-CMV, (Figure 1) vector were digested and purified by the same condition as product, but incubation time was 3 hr.

Next, these extracted DNAs were allowed to ligate. Ligation reaction was performed by vector-insert ratio of 1:10. Reaction mixture contained 0.5 pmol vector, 5 pmol insert, and 1 µl 10X ligation buffer, 3 units T4 lygase (Fermentas), and H_2_O to a total volume of 10 µl. Reaction mixture was incubated overnight in 16 °C. Ligation products were transformed into XlBlue competent cells by heat shock method. Transformed samples were inoculated onto 10-cm Petri dishes containing LB-agar plus 50 µg/ml of kanamycin. After 16–20 hr growth at 37 °C, 10–15 colonies were picked and grown in 4 ml of L-broth containing 50 µg/ml of kanamycin. Plasmids were extracted and were screened by reaction using pAd primer (5-ATAAgCAgAgCTggTTTAgTg-3) and HILr primer. reaction consisted of primary denaturation in 95 °C for 3 min followed by 30 cycles of denaturation at 95 °C for 30 sec, annealing at 50 °C for 30 sec, extension at 72°C for 45 s and a 7 min final extension at 72 °C. products were run on a 2% agarose gel, stained with EtBr, and then photographed under ultraviolet light. Inserts containing plasmids were used for homologous recombination.

***Generation of recombinant adenoviral plasmids by homologous recombination in E. coli***

Typically, 0.5–1.0 µg of a recombinant vector plasmid was linearized with *PmeI* (Promega), by conditions same as former digestions, purified by phenol, chloroform extraction and ethanol precipitation. Digestion products were added into 25 µl of electrocompetent *E. coli *BJ5183 cells and electroporation was performed in 2.0 mm cuvettes at 2,500 V, 200 ohms, and 25 mF in an Eppendorf Gene Pulser electroporator.

Electroporated cells were plated on Petri dishes containing L-agar plus 50 µg/ml of kanamycin. After 16–20 hr growth at 37 °C, the smaller colonies (which usually represented the recombinants) were picked and grown overnight in 4 ml of L-broth containing 50 µg/ml of kanamycin followed by minipreparation. Clones were first screened by analyzing their supercoiled sizes on agarose gel, comparing them to pAdEasy-1 as the control.

Recombinant clones were further tested by restriction endonuclease digestion with *PacI *(NEB). Digestion mixtures contained 20 μl products, 12 units of enzyme, 3 µl of the 10X 4 buffer, and H_2_O to a total volume of 30 μl. The reactions were allowed to proceed for 4 hr at 37 °C. Once confirmed, supercoiled plasmid was transformed into XlBlue cells for large-scale amplification of plasmid, followed by *PacI* enzyme digestion.

***Production of adenoviruses in mammalian cells***

Cells (0.5×10^6^ HEK293) were plated in 6-well plate 24 hr before transfection, by which time they reached 50–70% confluency. Cells were transfected with 0.8 µg of recombinant adenoviral vector , digested with *Pac*I and ethanol precipitated. Lipofectamin 2000 (Invitrogen) was applied according to the manufacturer’s instructions. Transfected cells were monitored for GFP expression and collected 7–10 days after transfection by scraping cells off flasks and pelleting them along with any floating cells in the culture. All but 3 ml of the supernatant was removed. After three cycles of freezing in liquid nitrogen and rapid thawing at 37 °C, 1 ml of viral lysate was used to infect 3–5 × 10^6^ HEK293 cells in a 25 cm^2^ flask. The efficiency of such infection could be conveniently followed with GFP. Three to four days later, viruses were harvested as described above. At this point, viral titers were often high enough to be used for gene transfer experiments in cultured cells.

## Results

***Recombinant adenovirus constructs production***

**Figure 2.**
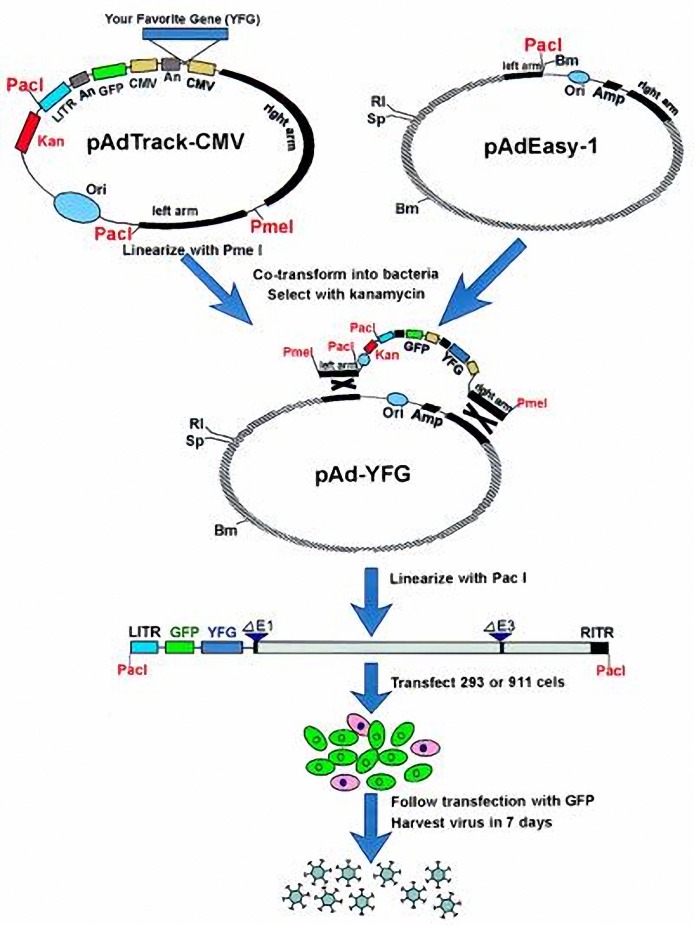
Schematic mechanism of the AdEasy system (17)

**Figure 3.**
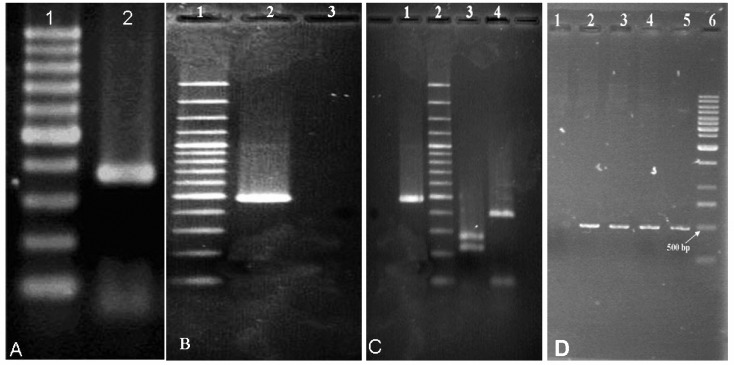
a) PCR product of PGM-1. Lane 1 is 100bp ladder and lane 2 is PGM-1 PCR product. b) product of IL-4: lane 1 is 100bp ladder and lane 2 is IL-4 PCR product, lane 3 is negative control. c) Restriction digestion of IL-4 PCR product. Lane 1 is intact IL-4 PCR product, lane 2 is 100 bp ladder, lane 3 is IL-4 digested with *PstI*, lane 4 is IL-4 digested with *EcoRV*.d) PCR results which were done by pAd primer. Lane 1is negative control, lane 2-5 are recombinant plasmids and lane 6 is 1Kb DNA ladder (Fermentas).

Figure 2 shows schematic mechanism of the AdEasy system (17). Figure 3a & 3b show RT-PCR results of PGM and IL-4. As Figures show the length of PGM1 and IL-4 products are 380 bp and 460 bp, respectively.

We initially digested IL-4 PCR products by *EcoRV* and *PstI* to PCR product identity confirmation, which its results have been shown in Figure 3c. According to Webcutter 2.0 online software analysis results (19), *EcoRV* cuts product in nucleotide 93 and *PstI* cuts it in 220 positions. Identity of products were confirmed by digestion results.

Both of IL-4 specific primers that have been used in this experiment have *XhoI* site in their 5' end, thus PCR product can get inserted into vector in both directions. In order to find recombinant vectors with correct insert orientation, PCR amplification was carried out using a primer complementary to upstream of vector multiple cloning site, pAd primer, as forward primer and HILr as reverse primer. Figure 3d shows results of recombinant plasmids. Observation of an approximately 500 bp band confirmed recombinant plasmid identity.

In the next step, we used homologous recombination between recombinant shuttle vector and pAdEasy-1 for production of recombinant virus construct.

Traditionally, two approaches have been used for generating recombinant adenoviruses. The first involves direct ligation of DNA fragments of the adenoviral genome to restriction endonuclease fragments containing a transgene. However, this method is ineffective due to the low efficiency of large fragment ligation. The second and more widely used method involves homologous recombination in mammalian cells capable of complementing defective adenoviruses (packaging lines). Homologous recombination results in a defective adenovirus that can replicate in the packaging line (e.g., 293 or 911 cells) supplying the missing gene products. However, this method is also challenging, because low efficiency of homologous recombination results in a need for repeated rounds of plaque purification from the mammalian cell line. This makes the method time-consuming and labor-intensive.

In this experiment, we used bacterial system to homologous recombination according to Tong-Chuan *et al* experiment with some modifications.

In Tong-Chuan *et al* experiment, shuttle vector (pAd-Track-CMV) and adenoviral vector are co-transformed into a bacterial host. This host is deficient in one of enzymes that mediate recombination (such as *recA*, *recB*, etc). BJ5138 strain that was used here is not *recA* deficient but is deficient in other enzyme of this process. This strain has been chosen because of its high efficiency of transformation. Co-transformation of intact Ad-Easy-1 and digested shuttle vector, leads to a reduction in recombinant colonies, because in optimum conditions, only one-fourth of electrocompetent cells have been transformed with both of the vectors. In order to solve this problem, we initially transformed BJ5138 cells by pAd-Easy-1 vector and chose ampicilin resistant colonies for electrocompetent production.

By recombination between these two plasmids, recombination occurs between right and left arms sequences of pAdEasy-1 and homologues sequences on pAd-Track-CMV. This leads to replacement of ori and Amp^r^ sequences of pAdEasy-1 by pAd-Track-CMV plasmid. The length of resulted construct approximately is equal with pAdEasy-1 and has similar electrophoretic pattern to pAdEasy-1. Clones with similar pattern to pAdEasy-1 were used for further analysis. In order to confirm recombinant construct, plasmids were digested by *Pac I* restriction enzyme. pAdEasy-1 has only one site for this enzyme between left and right arm sequences and digestion of plasmid by this enzyme gives an approximately 35 kb band. After recombination, this site is replaced by two sites, originated from shuttle vector, and digestion of recombinant plasmid by this enzyme gives two bands with approximately 32 and 5 kb in length.

**Figure 4.**
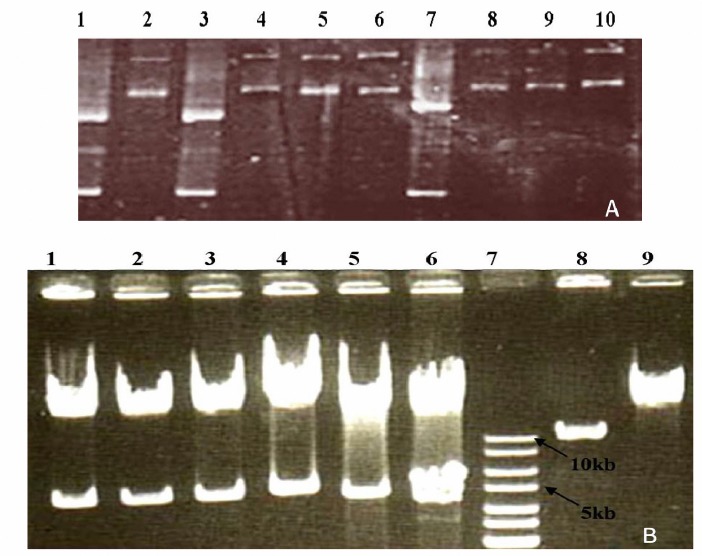
Electrophoretic pattern of electroporation results: lane 1 is a pAd-Track-CMV and lane 2 is an Ad-easy1 plasmid. Lanes 3-10 are electroporation results. According to controls, samples number 4-6 and 8-10 are possible viral constructs. b) Digestion of electroporation products by PacI: lane 1-6 are electroporation products, lane 7 is 1kb ladder and lanes 8 and 9 are pAd-Track and Adeasy, respectively.

Figure 4a shows electrophoretic pattern of plasmid DNA from different clones. *PacI* digestion results are shown in Figure 4b.

**Figure 5.**
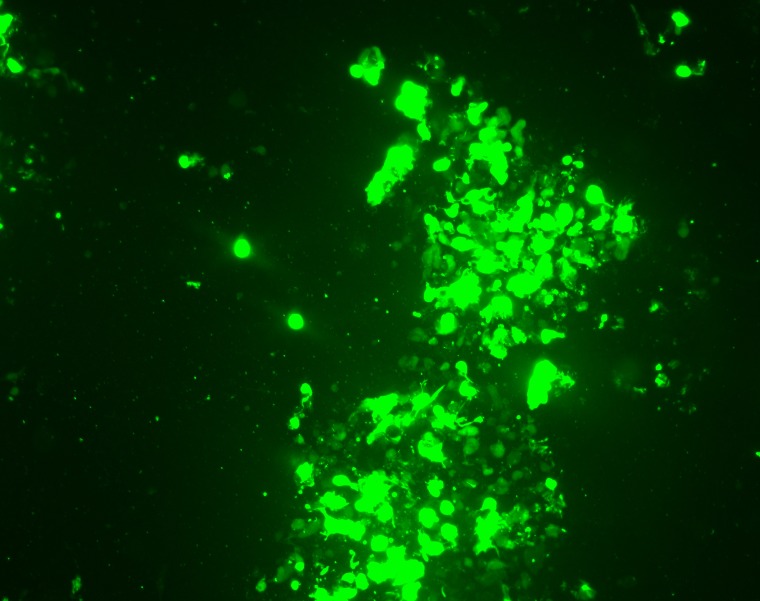
Virus producing HEK-293 cells. Green fluorescent protein (GFP) expression has seen as green color.

***Packaging of adenovirus in HEK293***

For packaging of adenovirus, recombinant adenoviral constructs containing IL-4 (designated Ad-IL4) must be cleaved by *PacI* restriction endonuclease. This exposes inverted terminal repeats of viral genome in packaging cell line.

Finally, viral genome must be transfected into HEK293 cell for packaging. Recombinant adenovirus is mutated in E3 and E4 genes which are essential for propagating of virus. HEK293 constitutively expresses these genes and adenovirus can be replicated and packaged in this cell line. Adenovirus producing cells appear under fluorescent microscopy, as fluorescent comet-like foci. 

In our experiment, these virus producing comet-like foci were appeared 5 days after transfection. 10 days after transfection up to 90% of cells expressed GFP that indicated virus production by cells. Figure 5 shows virus producing cells.

## Discussion

Recombinant adenovirus is one of the most efficient viral vectors for gene transfer, both in* vivo* and *in vitro*, due to its high transduction efficiency, broad host range, ability to infect non-dividing cells, and potential for generating high titer of virus. Its application is not just limited to gene therapy, but it can also be used as a tool in basic research to introduce transgenes into cells. For optimization of this method, we made a recombinant adenovirus that carries human IL-4 coding sequence and can express IL-4 in mammalian cells. 

Recent studies, clearly demonstrated that IL-4 has pleiotropic effects on immune cells of several such as dendritic cell (DC) maturation. This property, candidates IL-4 as a new agent for gene cancer therapy. 

Interestingly, IL-4 transduced cancer cells display increased lesional infiltration by DCs relative to other cytokines (20), which may result in enhanced cross-presentation of tumor-associated antigens by DCs.

Following these results, therapy of glioma by vaccination with autologous glioma cells engineered to produce IL-4 entered phase I clinical trials (21,22). In recent decades, several types of recombinant viruses have been made and used for various purposes, e.g., retrovirus for treatment of glioma and adenocarcinoma (23,24), and adenovirus for control of adjuvant-induced arthritis and glomerulonephritis (19, 25,26).

Furthermore, IL-4 is a cytokine with anti-inflammatory properties on activated macrophages. These characteristics of IL-4 have been employed for treatment of inflammatory and auto-immune diseases. Some studies focus on this field by using IL-4 protein for treatment of cells (27).

## Conclusion

Recombinant adenovirus that has been produced here may be used as an effective, easy, and cost benefit tool for studying immunity diseases and also gene therapy of some solid tumors.
